# The antimalarial drug quinine interferes with serotonin biosynthesis and action

**DOI:** 10.1038/srep03618

**Published:** 2014-01-09

**Authors:** Farida Islahudin, Sarah M. Tindall, Ian R. Mellor, Karen Swift, Hans E. M. Christensen, Kevin C. F. Fone, Richard J. Pleass, Kang-Nee Ting, Simon V. Avery

**Affiliations:** 1 School of Life Sciences, University of Nottingham, Nottingham NG7 2RD, UK; 2 School of Pharmacy, University of Nottingham Malaysia Campus, 43500 Semenyih, Malaysia; 3 Department of Chemistry, Technical University of Denmark, DK-2800 Kgs. Lyngby, Denmark; 4 University of Liverpool, Liverpool School of Tropical Medicine, Liverpool L3 5QA, UK; 5 School of Life Sciences, University of Nottingham Malaysia Campus, 43500 Semenyih, Malaysia; 6 Current address: Faculty of Pharmacy, University of Kebangsaan Malaysia, 50300, Kuala Lumpur, Malaysia.

## Abstract

The major antimalarial drug quinine perturbs uptake of the essential amino acid tryptophan, and patients with low plasma tryptophan are predisposed to adverse quinine reactions; symptoms of which are similar to indications of tryptophan depletion. As tryptophan is a precursor of the neurotransmitter serotonin (5-HT), here we test the hypothesis that quinine disrupts serotonin function. Quinine inhibited serotonin-induced proliferation of yeast as well as human (SHSY5Y) cells. One possible cause of this effect is through inhibition of 5-HT receptor activation by quinine, as we observed here. Furthermore, cells exhibited marked decreases in serotonin production during incubation with quinine. By assaying activity and kinetics of the rate-limiting enzyme for serotonin biosynthesis, tryptophan hydroxylase (TPH2), we showed that quinine competitively inhibits TPH2 in the presence of the substrate tryptophan. The study shows that quinine disrupts both serotonin biosynthesis and function, giving important new insight to the action of quinine on mammalian cells.

Quinine (QN) has been a mainstay drug in the battle against malaria, which is responsible for the loss of up to one million human lives each year^1^. However, QN efficacy as an antimalarial has been limited by some incidence of drug resistance^2^ and by common adverse reactions among patients^3^,^4^. Adverse reactions also impact the application of quinine as a treatment for leg cramps. The basis for these adverse reactions is poorly understood, but recent research has shed some new light on the issue. Studies using a model organism, the yeast *Saccharomyces cerevisiae*, revealed that transport of the essential amino acid tryptophan is inhibited by QN^5^. As a result, QN causes tryptophan starvation, a toxic mode of action that can be suppressed in media with high tryptophan^5^,^6^. The relevance of these findings to humans has been highlighted in clinical screens of malaria patients treated with QN^7^. This demonstrated that patients with lower plasma tryptophan were more likely to experience adverse reactions to QN, undermining its safety profile. This is important as tryptophan levels in humans vary considerably between individuals^8^,^9^. Such differences are at least partly due to diet and, therefore, dietary supplements could offer a simple way to manipulate QN responses in patients^7^.

This inter-relationship between QN and tryptophan resonates with an overlap in certain symptoms of tryptophan depletion and adverse QN reactions (cinochonism) in humans, such as tinnitus, anxiety and sleep disturbance^8^,^9^,^10^,^11^,^12^. The fact that neuropsychological effects are among these overlapping contra-indications is notable, as tryptophan is a metabolic precursor for the neurotransmitter serotonin (5-hydroxytryptamine, 5-HT). Indeed, tryptophan depletion results in decreased serotonin production^13^ and can trigger depression in susceptible individuals as discussed below.

The dysregulation of the monoamine serotonin in the brain has been extensively studied and has been linked to many psychiatric disorders^14^. Neuropsychological indications such as weakened decision making, poor mood or sleep, pain, appetite and depression have all been linked to serotonin function^14^,^15^,^16^. Serotonin in organisms is synthesised from tryptophan through two enzymatic reactions. The rate limiting reaction is catalysed by tryptophan hydroxylase (TPH), which converts tryptophan to the intermediate 5-hydroxytryptophan. Tryptophan hydroxylase-2 (TPH2) performs this function in the central nervous system, while another isoform, TPH1, does so in the periphery. Serotonin is subsequently produced from 5-hydroxytryptophan by aromatic amino acid decarboxylase. Serotonin production is dependent on tryptophan availability, which hinges partly on tryptophan transport across the blood-brain barrier^17^. Tryptophan is transported into the brain by the large neutral amino acid (LNAA) transporter, in competition with several other amino acids. Such interactions make tryptophan metabolism difficult to predict. However, perturbation of tryptophan availability has the potential to disrupt normal neuropsychological performance. For example, acute tryptophan depletion in patients with a history of depression and panic disorders can cause relapse of the disorder^18^. Tryptophan deficiency is common in malnourished populations in Africa, where QN is still routinely used as a treatment for severe malaria.

As QN affects tryptophan availability and patients with low tryptophan appear predisposed to adverse quinine reactions^5^,^7^, here we hypothesize that such adverse effects of the drug could reflect perturbation of serotonin function. It has been noted previously that QN interacts at 5-HT_3_ receptors^19^,^20^. We exploit the yeast model in conjunction with mammalian cells and biochemical assays to show that QN can inhibit both serotonin production and function.

## Results

### Quinine inhibits stimulation of yeast growth by serotonin

**Figure 1:**
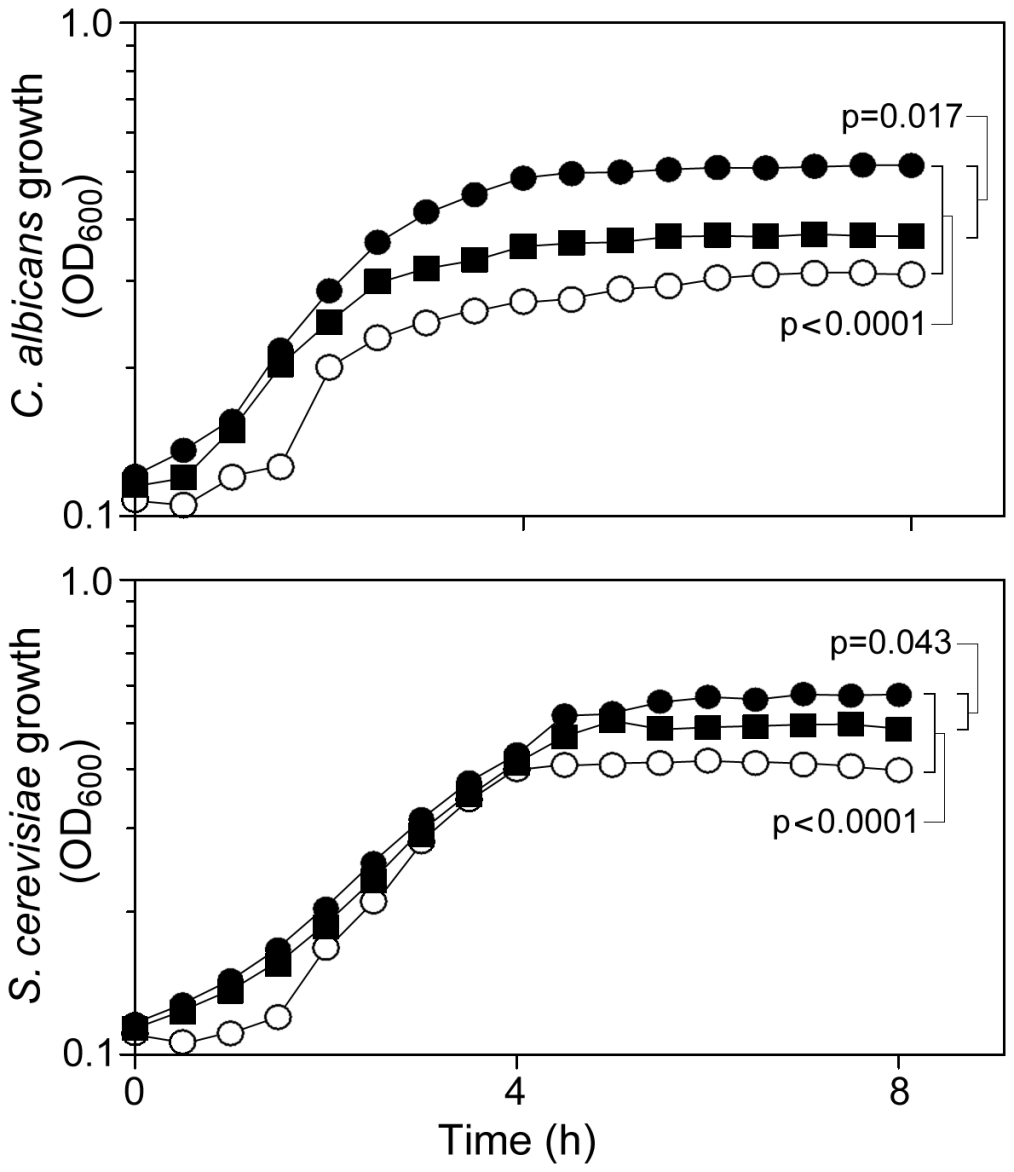
Quinine inhibits stimulation of yeast growth by serotonin. *C. albicans* SC5314 (upper panel) or *S. cerevisiae* BY4743 (lower) were cultured in unsupplemented Sabouraud's medium (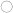) or medium supplemented with 1 mM serotonin (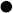), or 1 mM serotonin + 1 mM QN (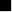). (This concentration of QN has no inhibitory effect on yeast growth when supplied alone^5^). Points are means from three independent experiments. SEMs were smaller than the dimensions of the symbols.

Previously it was shown that QN and tryptophan compete for uptake in yeast^5^, and elsewhere that the tryptophan derivative serotonin can stimulate yeast growth^21^. We exploited these insights in some preliminary tests for interference of serotonin action by QN, using the yeasts *C. albicans* and *S. cerevisiae*. The growth yield for *C. albicans* and *S. cerevisiae* was increased significantly in the presence of 1 mM serotonin, by 1.7-fold and 1.4-fold respectively (t-test, p < 0.0001 in both organisms) (Fig. 1). This indication of growth stimulation by serotonin was partly suppressed by the inclusion of 1 mM QN in the medium (t-test, p = 0.0017 and 0.0043 in *C. albicans* and *S. cerevisiae*, respectively). When supplied alone QN had no inhibitory effect on yeast growth at this concentration, as also reported previously^5^. Therefore, QN specifically blocked serotonin-stimulated growth.

### Quinine inhibits human type 2 serotonin-receptor activation

**Figure 2:**
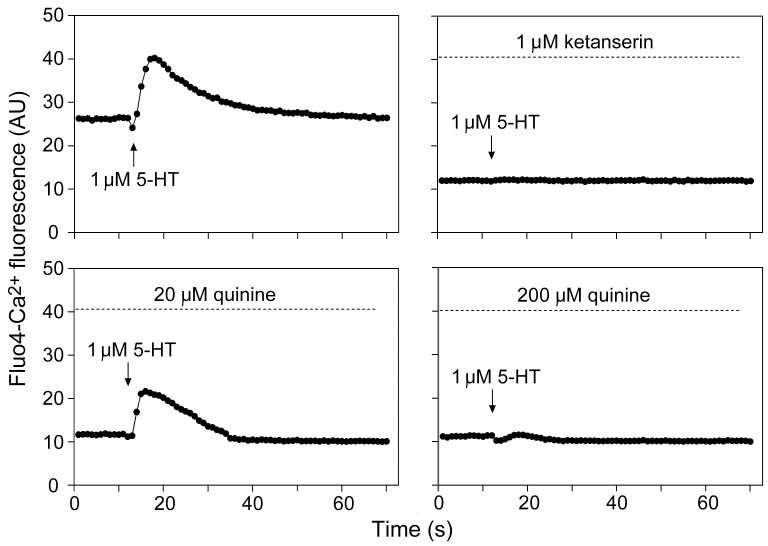
Quinine inhibits 5-HT_2_ receptor activation. Stimulation of calcium release from SHSY5Y cells by 1 μM serotonin addition was tested in the absence or presence of ketanserin or quinine at the indication concentrations. Mean data are shown from three independent experiments. AU, arbitrary units.

It was hypothesized that the inhibition by QN of serotonin action on yeast growth might reflect an interaction between these structurally-related molecules at one or more cell receptors. Previously, QN was found to block activation of heterologously-expressed mammalian 5-HT_3_ receptors^19^,^20^. To support and extend that finding, here the effect of QN was tested in a human neuroblastoma cell line (SHSY5Y) which expresses 5-HT_2a/2c_ receptors, by measuring the level of cellular calcium release in response to serotonin addition. Calcium release (traced with the FLUO-4 probe) was markedly stimulated by 1 μM serotonin (Fig. 2). The known potent 5-HT_2_ receptor antagonist, ketanserin, was used as a positive control. Ketanserin effectively blocked calcium release by serotonin addition (Fig. 2). At a concentration equimolar with serotonin, QN had negligible effect on receptor activation. However, at 20 μM QN, the response to serotonin was decreased by ~30% (t-test, p = 0.015) and there was no discernible response to serotonin in the presence of 200 μM QN.

### Quinine inhibits stimulation of neuroblastoma cell growth by serotonin

**Figure 3:**
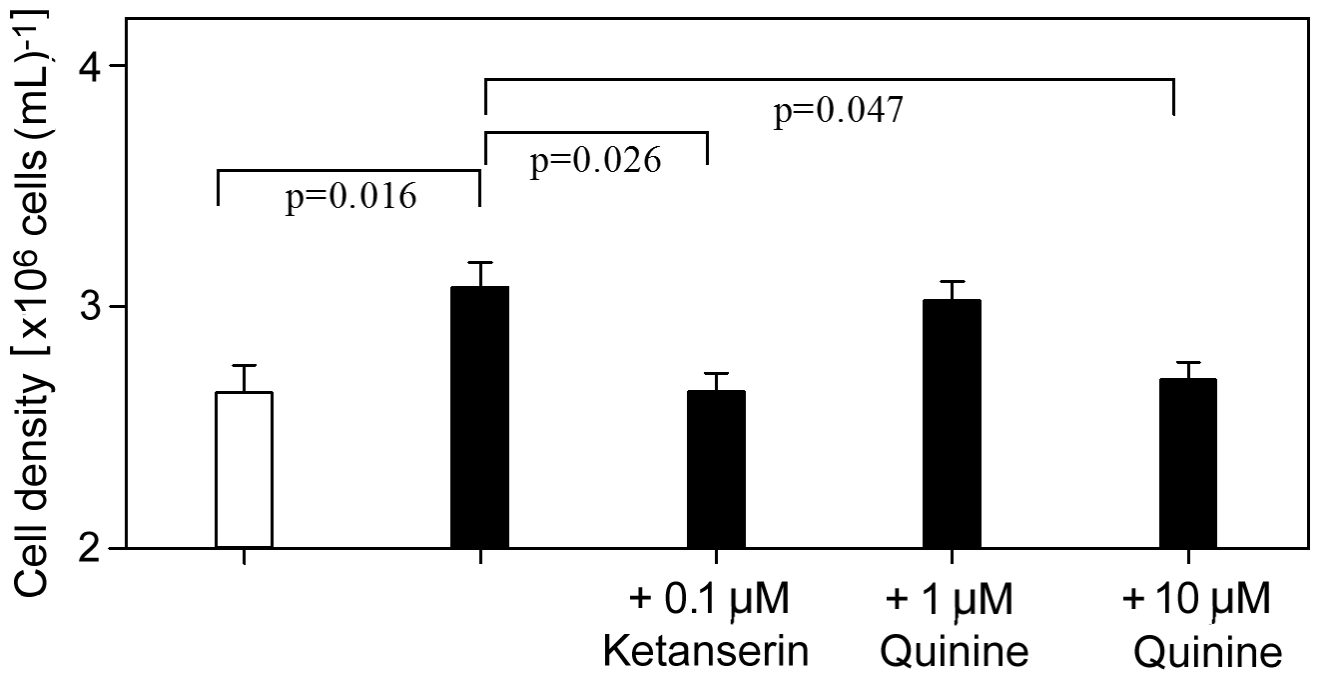
Quinine inhibits stimulation of neuroblastoma cell growth by serotonin. SHSY5Y cells were cultured in the absence (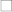) or presence (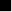) of 1 μM serotonin, with ketanserin or quinine supplied at the indicated concentrations where specified. Cell density was determined after 72 h incubation. Mean data ± SEM are shown from three independent experiments.

Having corroborated that QN interferes with serotonin receptors on mammalian cells, we sought to test whether the observed inhibition of serotonin-stimulated growth of yeast cells (Fig. 1) was also apparent in mammalian cells. Previously, low concentrations of serotonin have been reported to promote growth of tumorigenic human cells lines^22^. In the present work, it was found that 1 μM serotonin stimulated SHSY5Y cell proliferation by ~14% (t-test, p = 0.016) (Fig. 3). The inclusion of 0.1 μM ketanserin blocked this effect of serotonin. Similarly, and as in yeast, the presence of QN (10 μM) reversed the growth stimulatory effect of serotonin (Fig. 3). Neither 0.1 μM ketanserin nor 10 μM QN affected cell proliferation when supplied alone.

### Quinine inhibits serotonin production

**Figure 4:**
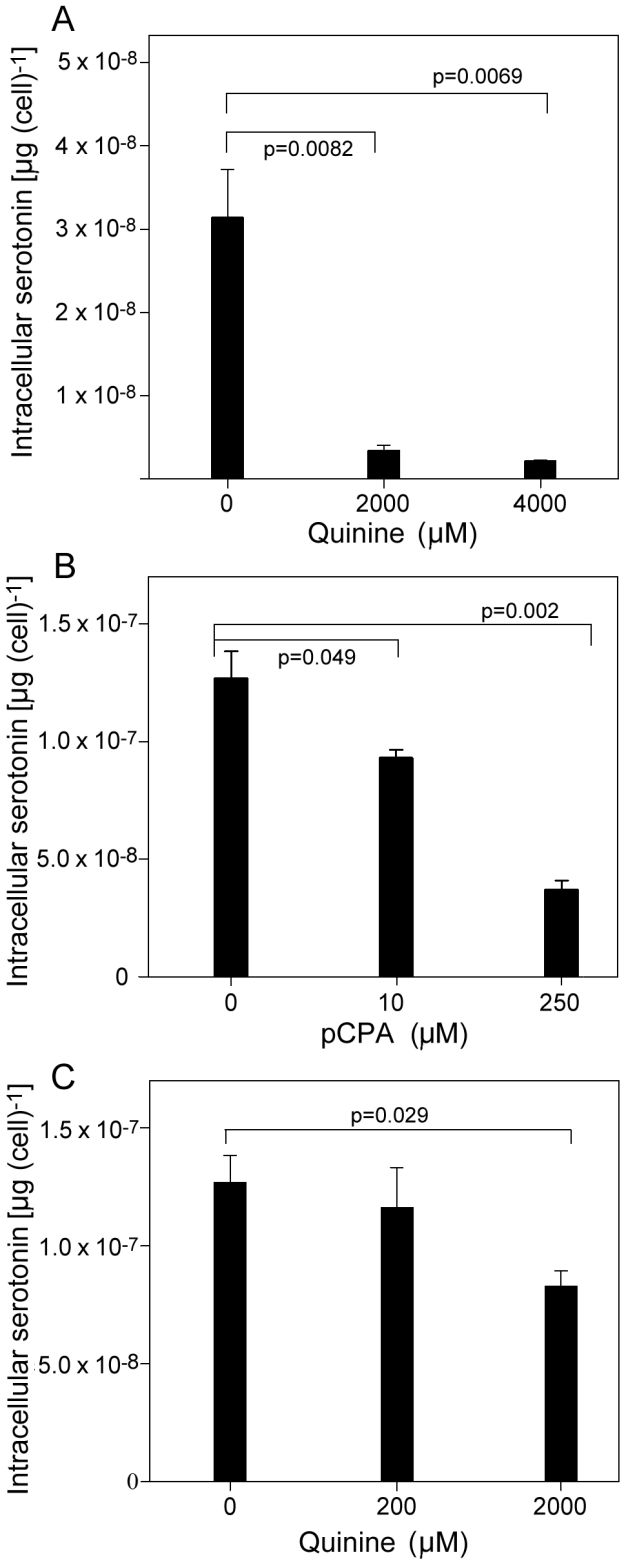
Quinine inhibits serotonin production. (A) Yeast cells transformed with pYES-TDC were cultured for 24 h in the presence of the indicated quinine concentration before determination of cellular serotonin with HPLC-ED. (B) Serotonin was determined in rat RN46A cells after 5 d culturing with the indicated concentration of the TPH2 inhibitor, pCPA. (C) Serotonin was determined in rat RN46A cells after 5 d culturing with the indicated concentration of quinine. Mean values are shown ± SEM from three independent experiments.

Having established that QN interferes with certain serotonin functions (above), we hypothesized that the antimalarial may also perturb serotonin biosynthesis as the pathway commences with tryptophan, with which QN competes^5^. Serotonin production was investigated first in yeast cells transformed with pYES-TDC. This plasmid harbours a tryptophan decarboxylase gene from rice, which produces serotonin from endogenous 5-hydroxy-tryptophan in yeast, so providing a yeast model for serotonin biosynthesis^23^. Determination of serotonin levels in the yeast cells by HPLC-ED showed that serotonin production was decreased significantly when cells were cultured in the presence of 2 mM (t-test, p = 0.0082) or 4 mM (p = 0.0069) QN (Fig. 4a). These concentrations were sub-inhibitory and mildly inhibitory to growth, respectively, but there was a ≥ 90% decrease in serotonin production in both cases.

The effect of QN on serotonin production was subsequently examined in the rat raphe RN46A cell line, which expresses TPH2 activity and naturally produces serotonin. As a positive control, the known TPH2 inhibitor, p-chlorophenylalanine (pCPA), was shown to decrease serotonin levels significantly at pCPA concentrations greater than 10 μM (Fig. 4b). In the case of QN, there was a decrease in cellular serotonin production with increased QN concentration. RN46A cells treated with 2 mM QN exhibited significantly lower serotonin levels, at 65% of the amounts determined in control cells (Fig. 4c). There was some growth inhibition of the RN46A cells at this QN concentration, but this was normalized by expressing serotonin amounts on a per cell basis.

### High levels of quinine uptake by RN46A cells are not affected by tryptophan supply

**Figure 5:**
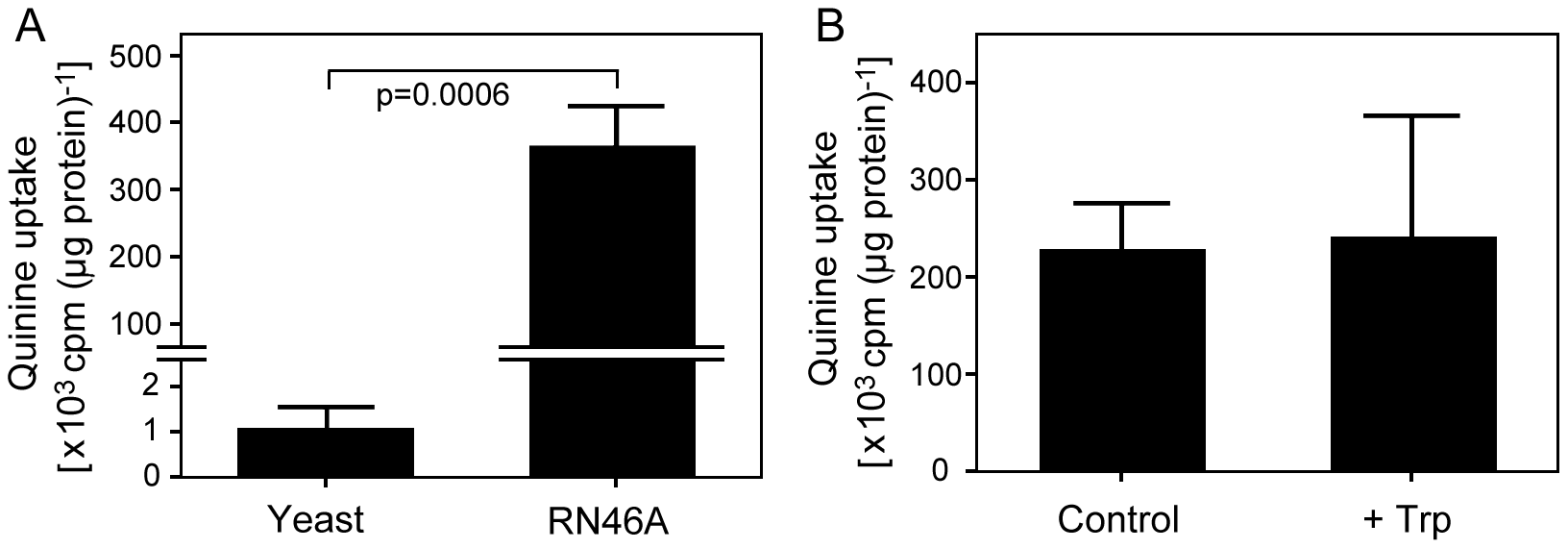
Quinine uptake by yeast and rat RN46A cells. (A) Yeast (transformed with pYES-TDC) or RN46A cells were incubated in the presence of 100 μM quinine supplemented with 0.9 μCi ml^−1^ [^3^H]-quinine. Cellular radiolabel uptake was determined after 24 h for yeast or 5 d for RN46A cells (i.e. the same intervals as for serotonin determination, Fig. 4) and the derived scintillation counts normalized to cellular protein. (B) As in (A) for RN46A cells, but with additional parallel incubations in the presence of 1 mM L-tryptophan. All values are means ± SEM from three independent experiments.

As QN inhibited serotonin production more strongly in yeast than in the rat RN46A cells (Fig. 4), we hypothesized that the yeast cells may take up more QN. To test this, [9-^3^H]-QN uptake was compared in the two cell types. Contrary to the hypothesis, QN uptake was much greater in the RN46A cells than in yeast (t-test, p = 0.006) (Fig. 5a). To explore QN uptake further in the rat cell line, the effect of excess tryptophan was measured. In contrast to previous work with yeast^5^, tryptophan addition did not affect QN uptake by RN46A cells (Fig. 5b) suggesting a distinct transport mechanism for QN in the rat cells.

### Quinine competitively inhibits tryptophan hydroxylase 2

**Figure 6:**
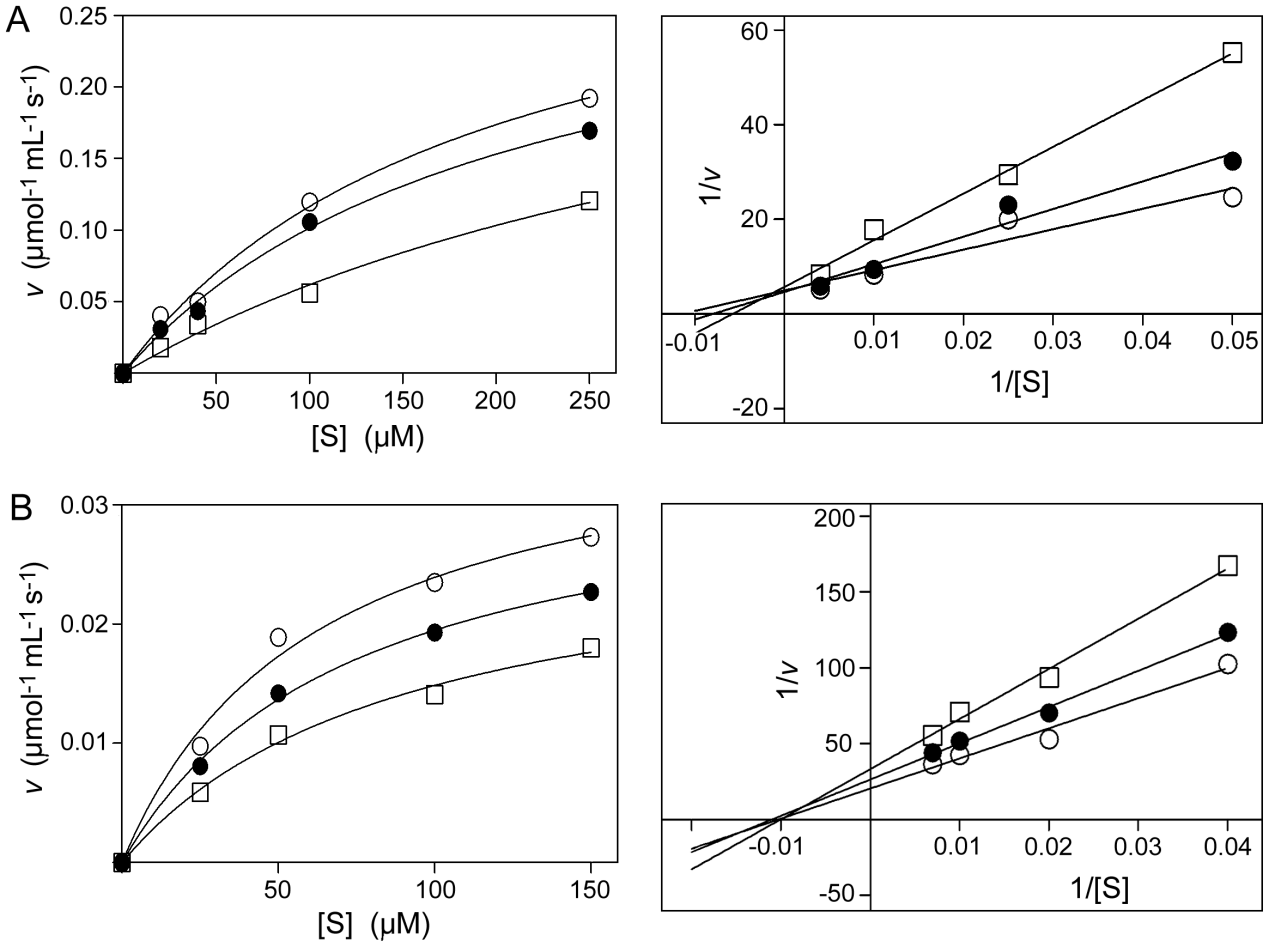
Effect of quinine on *in vitro* TPH2 activity. (A) Initial rate of activity of the purified catalytic domain of human TPH2 (*v*) was assayed in the presence of 0 (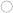), 2 (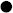) or 10 (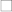) mM QN at different concentrations of the L-tryptophan substrate [S]. Points are means from three replicate determinations of *v.* SEMs were smaller than the dimensions of the symbols. The data are presented as substrate saturation curves (left panel) and Lineweaver-Burk plots (right). (B) As in (A) but where [S] refers to different concentrations of the TPH2 co-factor 6MePH_4_.

As the impact of QN on serotonin production is influenced by cell type (Fig. 4), and presumably also by *in vitro* experimental conditions for cells, we tested the action of QN directly on the main rate-limiting enzyme for serotonin biosynthesis, tryptophan hydroxylase 2 (TPH2). TPH2 catalyzes the synthesis of 5-hydroxytryptophan from tryptophan. The activity of the purified catalytic domain of human TPH2^24^ was assayed over a range of tryptophan (substrate) concentrations in the absence or presence of QN or pCPA. The data conformed to Michaelis–Menten kinetics, and revealed that increasing the concentration of QN from 0 mM to 2 mM then to 10 mM progressively decreased the TPH2 activity (Fig. 6a). Linearisation of the data to produce Lineweaver-Burk plots indicated that QN competitively inhibited TPH2 activity (as it was the intercept with the x axis (−1/*K_m_*) that was primarily affected by QN concentration). A similar outcome was obtained for the competitive-inhibitor of TPH2, pCPA, as a positive control (Supplementary Fig. S1a). The data indicate that, like pCPA, QN competes with tryptophan for binding to the active site of TPH2. The effects of QN and pCPA were also assayed over varying concentrations of a co-factor required for TPH2 activity, 6-methyltetrahydropterin (6MePH_4_). In this case, and in contrast to the interaction with tryptophan, altering the QN or pCPA concentrations gave non-competitive inhibition of (6MePH_4_-dependent) TPH2 activity (Fig. 6b and Supplementary Fig. S1b). This result was consistent with the role of 6MePH_4_ as the enzyme co-factor, and reinforced the distinctive competitive nature of the interaction between QN and the substrate tryptophan.

## Discussion

The key novel findings in this paper are that the antimalarial drug quinine can interfere with both production and function of the major neurotransmitter serotonin. This could help to explain certain adverse reactions to QN treatment seen among malaria patients, particularly those with low dietary tryptophan^3^,^4^,^7^. The results also raise the possibility that quinine could find application as an antidote against serotonin syndrome, a condition linked to excessive serotonin in patients^25^. As discussed below, the effect of QN on serotonin production could be attributable to competition between the drug and tryptophan (the substrate for serotonin biosynthesis) at two principal sites: the active site of the rate-limiting enzyme for serotonin biosynthesis (TPH), and transporters responsible for tryptophan uptake by cells.

To assay potential interactions between QN and serotonin function, we exploited previous reports of serotonin-induced cell proliferation in yeast and tumorigenic cells^21^,^22^,^26^,^27^, as well as the availability of 5-HT_2a,2c_ receptor-expressing cells. Aromatic alcohols act as autoinducers of yeast and tumorigenic cell growth. In nitrogen deficient media, tryptophol, an amino alcohol and tryptophan derivative is synthesized to autoinduce cell proliferation^21^. Because of the structural similarity between serotonin and tryptophol, serotonin can act as an autoinducer under the same conditions^21^. In the present study, QN suppressed these proliferative effects of serotonin. Amine alcohol receptors of yeast are poorly understood. In contrast, 5-HT receptors are well described in higher cells, including as therapeutic targets, and previous work indicated that QN inhibits activation of mammalian 5-HT_3_ receptors expressed in *Xenopus* oocytes or HEK-293 cells^19^,^20^. In addition, QN has been reported to inhibit active serotonin uptake into rat synaptosomes^28^ and to affect serotonin-modulated K^+^-channels^29^. Here, QN was observed to inhibit calcium signalling at 5-HT_2a/2c_ receptors. This is important as 5-HT_2_ receptors are linked to a variety of neuropsychological disorders such as anxiety and mood lowering effects^16^.

In mammals, serotonin production in the central nervous system is rate-limited by the TPH2 enzyme^30^. The present *in vitro* assays suggested that QN competes directly with tryptophan for binding to the active site of TPH2, similar to the known competitive TPH2 inhibitor, pCPA^31^. pCPA potently decreases serotonin production in the brain^31^. The inference that QN, similarly, may have the potential to suppress serotonin production by cells was borne out by analysis of serotonin levels in RN46A cells and, in particular, yeast cells. The strong effect in yeast cells was despite relatively low levels of QN uptake (Fig. 5a), highlighting the potential potency of QN action in inhibiting serotonin production. However, there was a smaller relative effect on serotonin production in the rat serotonergic cell line RN46A, despite much higher QN uptake. This indicates that the absolute intracellular QN level is not the sole factor that determines inhibition of serotonin production. We propose that another key factor involved here could be cellular tryptophan concentration. Previously we showed that QN and tryptophan compete for uptake via the Tat2p transporter in yeast, leading to tryptophan depletion^5^. The high level of QN uptake in RN46A cells appears to be through a different type of mechanism, as excess tryptophan did not affect QN uptake. This apparent lack of competition for uptake between tryptophan and QN suggests that QN is unlikely to cause the cellular tryptophan depletion in RN46A that occurs in yeast cells. Therefore, the lesser impact of cellular QN on serotonin production in RN46A cells may at least partly be attributable to relatively high tryptophan levels in these cells, as this would balance the competition for TPH2 binding in favour of the tryptophan substrate (Fig. 6a).

It is evident from the above discussion that the level of competition with tryptophan both for uptake into cells and for TPH2 binding may determine the relative impact of QN on serotonin production. Competition at the point of uptake, in particular, is likely to vary considerably: between cell types as seen here (e.g. depending on the transporters expressed by cells), and between *in vivo* physiological environments, as affected by interactions between cells, neurotransmitters and hormones as well as organ type. For example, the level of QN within the brains of patients is thought to be lower than in peripheral tissues due to the blood-brain barrier^32^. Such considerations may mitigate the fact that the effective QN concentrations used in certain of our *in vitro* experiments with the RN46A cells were higher than the recommended therapeutic dose, approaching QN concentrations that are toxic to mammalian cells. More to the point, the physiological relevance of interactions between QN and tryptophan has already been established in clinical studies, which provided evidence for competition between these molecules *in vivo* and showed that high plasma tryptophan decreases the incidence of adverse reactions to therapeutic doses of QN in malaria patients^7^. The present work shows how effects of QN on synthesis and function of the major tryptophan metabolite, serotonin, provides a potential explanation for such previous findings. This rationale has further indirect support, from the similarities in the reported adverse neuropsychological effects of QN and of serotonin imbalance, which include tinnitus, loss of appetite, sleep disturbance and anxiety^3^,^4^,^12^,^15^,^16^. These issues underscore how adherence to the narrow therapeutic index of QN during treatment may avert neurological toxicity and serious adverse effects. Even then, however, several of the effects we report (e.g., Figs. 2, 3, 5) did occur at quinine concentrations that are within the 4–100 μM ranges seen in human organs or plasma during quinine treatment^5^,^7^. The present work also leaves open the possibility that there are interactions between QN and tryptophan at cellular sites additional to those studied to date, which may have *in vivo* consequences beyond those suggested. For example, tryptophan is also a precursor in the kynurenine pathway, which is known to play a role in cerebral malaria^33^,^34^. A high level of QN uptake by mammalian cells, indicated here, may underpin many effects of this drug.

## Methods

### Yeast growth

The yeast strains *Saccharomyces cerevisiae* BY4743 or *Candida albicans* SC5314 were cultured in Sabouraud's medium containing 30 g L^−1^ maltose, 10 g L^−1^ peptone from casein (Sigma), 3 g L^−1^ yeast extract (Sigma), adjusted to pH 7.0^21^. After 24 h growth of starter-cultures, strains were inoculated to the same medium in Erlenmeyer flasks and incubated for 4 h at 34°C with orbital shaking at 120 rev min^−1^. Cultures were then adjusted to 10^6^ cells mL^−1^ in the same medium and distributed in 300 μL aliquots per well in 48-well plates (Greiner Bio-One). Wells were supplemented with serotonin hydrochloride (Sigma) or quinine dihydrochloride dihydrate (QN; Sigma) as specified. Growth of the yeasts with shaking at 34°C was monitored with a BioTek Power Wave XS2 Microplate Spectrophotometer.

### Mammalian cell culture

Human neuroblastoma SHSY5Y cells (a gift from Professor David Kendall, University of Nottingham) and rat raphe RN46A cells (a gift from Dr Scott Whittemore, University of Louisville) were cultured in DMEM-F12 HAM nutrient medium (Sigma) supplemented with 10% (v/v) fetal calf serum (Sigma), 2 mM L-glutamine, 100 U mL^−1^ penicillin and 100 μg mL^−1^ streptomycin^35^. Cells were cultured in 25 cm^2^ cell culture flasks in 5 mL DMEM-F12 medium at 36.5°C, 5% CO_2_ until confluent. Cells were detached from the bases of flasks with 2 mL trypsin-EDTA solution (Sigma). DMEM-F12 medium (10 mL) was added to the flask, followed by 7 min centrifugation at 600 *g*. The cell pellet was resuspended in 10 mL DMEM-F12. An aliquot (0.5 mL) of the cell suspension was added to 4.5 mL DMEM-F12 in 25 cm^2^ cell culture flasks, and cultured until confluent at 36.5°C in 5% CO_2_.

### Mammalian cell proliferation

The assay was adapted from that described previously^22^. From confluent cultures (above), SHSY5Y cells were adjusted to 5 × 10^5^ cells mL^−1^ in DMEM-F12 medium and 100 μL aliquots seeded to individual wells of flat-bottomed 96-well plates. Wells were supplemented with serotonin, ketanserin (a selective 5-HT_2_ receptor antagonist, Sigma) or QN as specified in the results section. Plates were incubated at 36.5°C, 5% CO_2_ for 72 h, before cells were detached by addition of 50 μL trypsin-EDTA solution. Detached cell mixture was added to 200 μL fresh medium in microcentrifuge tubes. Cells were centrifuged (600 *g*, 7 min) and the supernatant removed by aspiration. Cells were resuspended in 100 μL fresh medium and counted using a Neubauer hemocytometer (1/400 square mm, depth 0.1 mm).

### Serotonin receptor activity

SHSY5Y cells were cultured in clear-bottomed 96-well black plates (Cellstar) for 48 h in DMEM-F12 at 36.5°C, 5% CO_2_. The medium was then aspirated and 200 μL of DMEM-F12, 10% (v/v) fetal calf serum, containing 100 U mL^−1^ penicillin, 100 μg mL^−1^ streptomycin, 1 μM FLUO-4 (Invitrogen) and 2.5 mM probenecid (Sigma), were aliquoted to each well. After 45 min incubation at 30°C, 5% CO_2_, cells were washed with HEPES buffered saline (10 mM HEPES, 2 mM sodium pyruvate, 10 mM D-glucose, 0.145 M sodium chloride, 1 mM magnesium sulphate, 5 mM potassium chloride, 1.3 mM calcium chloride, 1.5 mM sodium hydrogen carbonate, pH 7.45) containing 2.5 mM probenecid. Following this, 180 μL of HEPES buffered saline containing 2.5 mM probenecid were added to each well. Where specified, the saline was supplemented with ketanserin or QN. After incubation of plates on a hot plate at 30°C for 20 min, the cells were examined with a FlexStation3 Microplate Reader (Molecular Devices) using SoftMax Pro software, with fluorescence from the FLUO-4 probe determined at 485 nm excitation and 525 nm emission. Where specified, serotonin (20 μL) was added to a final concentration of 10^−5^ M.

### Determination of cellular serotonin

For determinations in *S. cerevisiae* BY4743, cells were transformed with plasmid pYES-TDC^23^, using the lithium acetate method^36^. Transformant cells were cultured to OD_600_ ~ 2.0 in YNB broth with appropriate selection^37^ before sub-culturing to fresh medium supplemented with or without QN. Cells were harvested for analysis after 24 h incubation at 30°C. For RN46A cells, these were seeded from confluent flasks (see above) to fresh medium supplemented with QN or p-chlorophenylalanine (pCPA; Sigma) and incubated for 5 d at 36.5°C, 5% CO_2_. After harvesting by centrifugation, cells were suspended in 0.5 mL (yeast) or 1 mL of 0.1 M perchloric acid containing 1.6 mM sodium metabisulphite. Cell lysis of yeast cells was facilitiated by mechanical breakage with glass beads using a Fast Prep-24 MP bead beater. Cell lysates were centrifuged for 3 min at 15000 *g* at 4°C, and supernatants were stored at −80°C until analysis. Protein was determined with a Bradford assay kit (BioRad). Serotonin was determined using high performance liquid chromatography with electrochemical detection (HPLC-ED) as previously described^38^. In brief, samples were thawed, weighed and sonicated (Soniprep 150: MSE Scientific Instruments; Crawley, UK) for 30 s in 800 μL of 0.05 M perchloric acid containing 1 μM sodium metabisulphite, then centrifuged (17,400 *g*, 4°C for 20 min; Harrier 18/80: MSE Scientific Instruments; Crawley, UK) and the supernatant filtered (0.45 μm syringe tip filter, Kinesis Ltd; St Neots, UK). Monoamines were separated using a PerkinElmer Series 200 autosampler and Targa C18 3 μm column (100 mm × 2.1 mm: Presearch; Basingstoke, UK) and detected at a potential of +0.59 V against ISAAC reference electrode by an Antec Intro amperometric detector (Zoeterwoude, The Netherlands). Mobile phase consisting of 20 mM potassium dihydrogen orthophosphate, 20 mM sodium acetate, 8 mM potassium chloride, 0.1 mM disodium ethylenediaminetetraacetic acid (EDTA), 0.16 mM octanesulphonic acid and 10% v/v methanol adjusted to pH 3.8–4 was delivered at 0.2 mL min^−1^ by an isocratic pump (Dionex P680) and quantification used Galaxie (version 1.8) software. (Antec). The lower detection limit was 1 pmol per 20 μL sample.

### Quinine uptake

*S. cerevisiae* BY4743 transformed with plasmid pYES-TDC, or RN46A cells were cultured as described above. Media were supplemented with 0.1 mM QN, and [9-^3^H]-QN (American Radiolabeled Chemicals) to a final activity of 0.9 μCi ml^−1^ [10–20 Ci (mmol QN)^−1^]. Where indicated, L-tryptophan was added to a final concentration of 1 mM. After the same incubation times with QN as used above for determination of cellular serotonin (24 h for yeast, 5 d for RN46A), cells were washed and resuspended in phosphate buffered saline before splitting aliquots for transfer to scintillation vials or retention for protein determination. Cell-associated radioactivity was quantified in a liquid scintillation counter (2250CA Packard Tri-carb) and the readings normalized to protein content, determined as described above.

### TPH2 activity

A purified catalytic domain of human TPH2^24^ was assayed for activity as described previously^24^,^39^. The assay buffer comprised 200 mM ammonium sulphate, 7 mM DTT, 25 μg mL^−1^ catalase, 25 μM ferrous ammonium sulphate, 50 mM MES, pH 7.0, 150 μM 6-methyltetrahydropterin (6MePH_4_) (Sigma), 25 nM TPH2 enzyme and 20 μM tryptophan. Where specified, tryptophan (0.02–0.25 mM) or 6MePH_4_ (0.025–0.15 mM) concentrations were varied, and/or the assay buffer was supplemented with QN. Enzyme activity was quantified with fluorescence spectrophotometry (Cary Eclipse Varian) using a 4 mm quartz cuvette, 300 nm excitation, 330 nm emission, with a 5 nm band pass.

## Author Contributions

F.I. and S.M.T. performed the experiments. F.I., S.M.T., I.R.M., R.J.P., K.N.T. and S.V.A. designed the experiments. K.S. performed the serotonin analyses. F.I., K.N.T. and S.V.A. prepared the manuscript. I.R.M., K.C.F.F., H.E.M.C. and R.J.P. provided valuable materials and conceptual input and also reviewed the manuscript.

## Supplementary Material

Supplementary Information Supplementary Information Figure S1.
