# Supplementary material for: The antimalarial drug quinine interferes with serotonin biosynthesis and action
Source: Sci Rep. 2014 Jan 9;4:3618. doi: 10.1038/srep03618 (PMC3885885; doi:10.1038/srep03618)
Supplement: Supplementary Information — Figure S1. [file srep03618-s1.pdf]

Supplementary Information for The antimalarial drug quinine interferes with serotonin biosynthesis and action

Farida Islahudin, Sarah Tindall, Ian R. Mellor, Karen Swift, Hans E.M. Christensen, Kevin C.F. Fone, Richard J. Pleass, Kang-Nee Ting, Simon V. Avery

A.

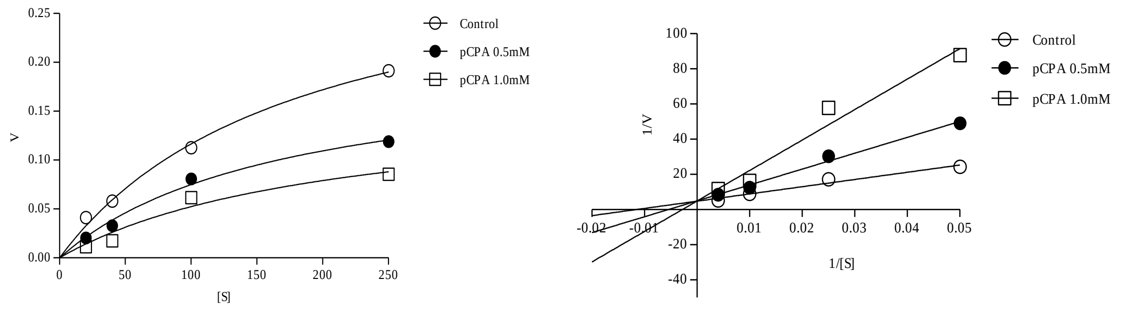

B.

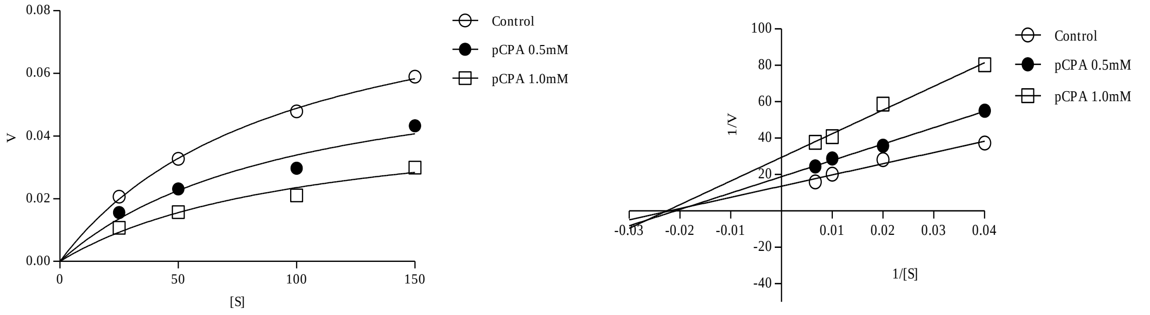

Supplementary Information Figure S1. Effect of pCPA on *in vitro* TPH2 activity. (A) Initial rate of activity of the purified catalytic domain of human TPH2 (*v*) was assayed in the presence of 0 (○), 0.5 (●) or 1.0 (□) mM pCPA at different concentrations of the L-tryptophan substrate [S]. Points are means from three replicate determinations of *v*. SEMs were smaller than the dimensions of the symbols. The data are presented as substrate saturation curves (left panel) and Lineweaver-Burk plots (right). (B) As in (A) but where [S] refers to different concentrations of the TPH2 co-factor $6MePH_4$.
